# Efficacy and safety with ticagrelor in patients with prior myocardial infarction in the approved European label: insights from PEGASUS-TIMI 54

**DOI:** 10.1093/ehjcvp/pvz020

**Published:** 2019-06-20

**Authors:** Mikael Dellborg, Marc P Bonaca, Robert F Storey, P Gabriel Steg, Kyung A Im, Marc Cohen, Deepak L Bhatt, Ton Oude Ophuis, Andrezej Budaj, Christian Hamm, Jindrich Spinar, Robert G Kiss, José Lopez-Sendon, Gabriel Kamensky, Frans Van de Werf, Diego Ardissino, Frederic Kontny, Gilles Montalescot, Per Johanson, Olof Bengtsson, Anders Himmelmann, Eugene Braunwald, Marc S Sabatine

**Affiliations:** 1 Department of Medicine, University of Gothenburg, Institute of Medicine, Sahlgrenska Academy, Sahlgrenska University Hospital/Östra, Diagnosvägen 11, Gothenburg, Sweden; 2 Harvard Medical School, TIMI Study Group, Boston, MA, USA; 3 University of Sheffield, Sheffield, UK; 4 University Paris Diderot, INSERM Unite 1148, Hôptial Bichat, Paris, France; 5 Newark Beth Israel Medical Center, Rutgers New Jersey Medical School, Newark, NJ, USA; 6 Canisius-Wilhelmina Hospital, Nijmegen, the Netherlands; 7 Postgraduate Medical School, Grochowski Hospital, Warsaw, Poland; 8 Kerckhoff Heart Center, Bad Nauheim, University of Giessen, Giessen, Germany; 9 University Hospital, Jihlavska, Brno, Czech Republic; 10 Department of Cardiology, Military Hospital, Budapest, Hungary; 11 Cardiovascular Division, University Hospital La Paz, Madrid, Spain; 12 Department of Noninvasive Cardiovascular Diagnostics, Vth Internal Clinic, University Hospital Bratislava, Bratislava, Slovakia; 13 University of Leuven, Leuven, Belgium; 14 Division of Cardiology, Azienda Ospedaliero Universitaria di Parma, Parma, Italy; 15 Department of Cardiology, Stavanger University Hospital, Stavanger, Norway; 16 Drammen Heart Center, Drammen, Norway; 17 Sorbonne Université Paris 6, ACTION Study Group, INSERM-UMRS 1166, Institut de Cardiologie, Pitié-Salpêtrière Hospital (AP-HP), Paris, France; 18 AstraZeneca, Mölndal, Sweden

**Keywords:** Dual antiplatelet treatment, Coronary artery disease, Post-myocardial infarction

## Abstract

**Aims:**

In PEGASUS-TIMI 54, ticagrelor significantly reduced the risk of the composite of major adverse cardiovascular (CV) events by 15–16% in stable patients with a prior myocardial infarction (MI) 1–3 years earlier. We report the efficacy and safety in the subpopulation recommended for treatment in the European (EU) label, i.e. treatment with 60 mg b.i.d. initiated up to 2 years from the MI, or within 1 year after stopping previous adenosine diphosphate receptor inhibitor treatment.

**Methods and results:**

Of the 21 162 patients enrolled in PEGASUS-TIMI 54, 10 779 patients were included in the primary analysis for this study, randomized to ticagrelor 60 mg (*n* = 5388) or matching placebo (*n* = 5391). The cumulative proportions of patients with events at 36 months were calculated by the Kaplan–Meier (KM) method. The composite of CV death, MI, or stroke occurred less frequently in the ticagrelor group (7.9% KM rate vs. 9.6%), hazard ratio (HR) 0.80 [95% confidence interval (CI) 0.70–0.91; *P* = 0.001]. Ticagrelor also reduced the risk of all-cause mortality, HR 0.80 (0.67–0.96; *P* = 0.018). Thrombolysis in myocardial infarction major bleeding was more frequent in the ticagrelor group 2.5% vs. 1.1%; HR 2.36 (1.65–3.39; *P* < 0.001). The corresponding HR for fatal or intracranial bleeding was 1.17 (0.68–2.01; *P* = 0.58).

**Conclusion:**

In PEGASUS-TIMI 54, treatment with ticagrelor 60 mg as recommended in the EU label, was associated with a relative risk reduction of 20% in CV death, MI, or stroke. Thrombolysis in myocardial infarction major bleeding was increased, but fatal or intracranial bleeding was similar to placebo. There appears to be a favourable benefit-risk ratio for long-term ticagrelor 60 mg in this population.

**Clinical trial registration:**

http://www.clinicaltrials.gov NCT01225562

## Introduction

In acute coronary syndrome with or without ST-segment elevation, European guidelines recommend dual antiplatelet treatment for at least the first year.^1^^,^^2^ Notably, many stable patients with a history of myocardial infarction (MI) remain at high risk after this period.^3^^,^^4^ In PEGASUS-TIMI 54, ticagrelor, at doses of either 90 mg b.i.d. or 60 mg b.i.d., significantly reduced the risk of the composite of major adverse cardiovascular events [MACE; cardiovascular (CV) death, MI, or stroke] by 15–16% in stable patients at high risk with a prior MI 1–3 years earlier.^5^ The benefit of ticagrelor appeared more marked in patients continuing on or restarting after only a brief interruption of adenosine diphosphate (ADP) receptor inhibition and in those closer to their qualifying MI.^6^ Accordingly, the CHMP-EMA approved European (EU) label recommends that, after the initial 1-year treatment with ticagrelor 90 mg b.i.d. (or other ADP receptor inhibitor) in high-risk MI patients, treatment with ticagrelor 60 mg b.i.d. may be started without interruption as continuation therapy.^7^ Treatment with ticagrelor 60 mg b.i.d. can also be initiated up to 2 years from the MI, or within 1 year after stopping previous ADP receptor inhibitor treatment. While the PEGASUS-TIMI 54 trial had wider inclusion criteria, the present analysis aimed to describe the effects of extended treatment with ticagrelor 60 mg b.i.d. in a clinically relevant subset of patients, treated according to the approved label. We, therefore, report the efficacy and safety in the PEGASUS-TIMI 54 subpopulation recommended for treatment in the EU label.

## Methods

### Study population

PEGASUS-TIMI 54 randomized patients with prior MI to ticagrelor 60 mg b.i.d., ticagrelor 90 mg b.i.d., or placebo, all on a background of low-dose aspirin. The protocol was approved by the relevant ethics committee at each participating site. Written informed consent was obtained from all the patients. The design^8^ and primary results of the trial have been published.^5^ In brief, patients aged at least 50 years were included with a spontaneous MI occurring 1–3 years prior to enrolment and at least one of the following additional high-risk features: age of 65 years or older, diabetes mellitus requiring medication, a second prior spontaneous MI, multivessel coronary artery disease, or chronic renal dysfunction, defined as a creatinine clearance <60 mL/min as estimated by the Cockroft–Gault equation. Patients were ineligible if there was planned use of a P2Y_12_ receptor antagonist or anticoagulant therapy during the study period; if they had a bleeding disorder, a history of intracranial bleeding, a central nervous system tumour, or an intracranial vascular abnormality; or if they had had gastrointestinal bleeding within the previous 6 months or major surgery within the previous month.

The present analysis focuses on data from 10 779 patients that were randomized ≤2 years from qualifying MI or ≤1year from prior stopping ADP receptor inhibitor treatment, 5388 in the ticagrelor 60 mg and 5391 in the placebo group (EU label group). Patients randomized to ticagrelor 60 mg or placebo who did not qualify per the EU label are termed the non-EU label group. Data on patients randomized to ticagrelor 90 mg who would have qualified for the EU label (*n* = 5374) are also presented in the Supplementary material online for the sake of completeness.

### Endpoints

The primary efficacy endpoint for PEGASUS-TIMI 54 was the composite of CV death, MI, or stroke (MACE). Additional efficacy endpoints included the individual components of the composite as well as coronary heart disease-related death and all-cause mortality. The primary safety endpoint was Thrombolysis in myocardial infarction (TIMI) major bleeding. Other safety endpoints included TIMI minor bleeding, intracranial haemorrhage, and fatal bleeding. All potential events were adjudicated by the TIMI clinical events committee, which was blinded to treatment allocation. Net clinical benefit was calculated as the number of events prevented (CV death, MI, stroke, or the composite of these) vs. events caused (TIMI major bleeding, intracranial haemorrhage, or fatal bleeding) per 1000 patients treated for 3 years with ticagrelor. Within these events we also examined the irreversible hard outcomes which included all of the aforementioned outcomes except TIMI major bleeding.^9^

### Statistical considerations

Cumulative event rates at 3 years were calculated by the complement of the Kaplan–Meier (KM) survival estimates. Hazard ratios (HRs) and 95% confidence intervals (CIs) were generated with the use of a Cox proportional-hazards model, and all reported *P*-values are two-sided. Interactions between the agreed EU label and the treatment group were also examined by Cox proportional hazards model. The assumption of proportional hazards was tested by including time dependent covariates in the model and examined by scaled Schoenfeld residual plots. Number needed to treat (NNT) was calculated by the reciprocal of the absolute risk difference based on 3 year KM estimates. The number of events prevented and caused per 1000 patients were based on the difference between 3 years incidence rates/person years in the treatment and placebo arm, with negative difference being ‘prevented’ events and positive difference being events ‘caused’ by treatment arm. This difference was multiplied by 1000 to aid the clinical interpretation. Efficacy analyses were performed on an intention-to-treat basis. Safety analyses included all the patients who received at least one dose of study drug and included all the events occurring after receipt of the first dose and within 7 days of the last dose of study drug. The bleeding analysis is on-treatment. Results for the 90 mg b.i.d. dose are presented in the Supplementary material online.

All analyses were performed according to the intention-to-treat principle, utilizing SAS version 9.4. The statistical significance was set at an α-level of 0.05 significance.

## Results

A total of 14 112 patients were randomized to ticagrelor 60 mg bid (the EU dose approved for long-term therapy) or placebo. Of this group, 10 799 patients were within 2 years from qualifying MI or within 1 year from prior ADP receptor inhibitor treatment and were randomized to ticagrelor 60 mg bid or placebo. As expected, there were no differences in baseline characteristics by randomized treatment arm (*Table 1*). An additional 3333 patients were in the ticagrelor 60 mg or placebo arms but fell outside the EU label parameters. The median time from MI was 1.5 years vs. 2.5 years and median time from P2Y_12_-treatment discontinuation was 34 days vs. 588 days for the EU label vs. non-EU label patients, respectively. Compared with non-EU label patients, EU label patients were more likely to have had a history of multivessel coronary artery disease (61.4% vs. 53.7%, *P* < 0.001) and a history of percutaneous coronary intervention (PCI) (84.9% vs. 77.1%, *P* < 0.001) (Supplementary material online, *Table S1*).

**Table 1:** Baseline characteristics ticagrelor 60 mg and placebo, European label and non-European label population

Characteristics	EU label population		Non-EU label population	
	Ticagrelor 60 mg bid (*N* = 5388)	Placebo (*N* = 5391)	Ticagrelor 60 mg bid (*N* = 1657)	Placebo (*N* = 1676)
Age (years), mean (SD)	65.1 (8.5)	65.3 (8.3)	65.5 (8.1)	65.6 (8.2)
Female	1267 (23.52%)	1314 (24.37%)	394 (23.78%)	403 (24.05%)
White	4592 (85.23%)	4606 (85.44%)	1485 (89.62%)	1518 (90.57%)
Weight, mean (SD)	81.9 (17.1)	81.6 (16.8)	82.5 (16.6)	82.5 (16.0)
History of hypertension	4183 (77.65%)	4175 (77.44%)	1278 (77.13%)	1309 (78.1%)
History of hypercholesterolaemia	4122 (76.52%)	4179 (77.52%)	1258 (75.97%)	1272 (75.94%)
Current smoker	939 (17.43%)	865 (16.06%)	267 (16.11%)	278 (16.59%)
History of diabetes	1774 (32.93%)	1710 (31.72%)	534 (32.25%)	547 (32.64%)
Multivessel coronary artery disease	3313 (61.5%)	3300 (61.21%)	877 (52.99%)	913 (54.47%)
History of PCI	4584 (85.09%)	4563 (84.66%)	1295 (78.15%)	1274 (76.01%)
History of second prior MI	884 (16.41%)	900 (16.69%)	284 (17.15%)	288 (17.18%)
History of PAD	301 (5.59%)	317 (5.88%)	67 (4.04%)	87 (5.19%)
eGRR <60 mL/min/1.73 m^2^	1178 (22.16%)	1239 (23.25%)	369 (22.51%)	410 (24.77%)
Qualifying event				
Years since MI, median (IQR)	1.5 (1.2–1.9)	1.5 (1.2–1.9)	2.5 (2.3–2.8)	2.5 (2.3–2.8)
Type of MI				
NSTEMI	2209 (41.04%)	2177 (40.43%)	633 (38.32%)	666 (39.81%)
STEMI	2872 (53.35%)	2928 (54.38%)	885 (53.57%)	881 (52.66%)
Unknown	302 (5.61%)	279 (5.18%)	134 (8.11%)	126 (7.53%)
Medications at baseline				
Aspirin	5381 (99.87%)	5382 (99.83%)	1655 (99.88%)	1675 (99.94%)
Statin	4999 (92.78%)	5049 (93.66%)	1496 (90.28%)	1534 (91.53%)
Beta blocker	4462 (82.81%)	4518 (83.81%)	1334 (80.51%)	1360 (81.15%)
ACE-I or ARB	4310 (79.99%)	4341 (80.52%)	1321 (79.72%)	1356 (80.91%)

There were no statistically significant differences in baseline characteristics by treatment arm within the EU and non-EU label subgroups.

In addition, there were 5374 patients randomized to ticagrelor 90 mg within 2 years from qualifying MI or within 1 year from prior ADP receptor inhibitor treatment (see Supplementary material online, *Table S2*).

### Efficacy

**Figure 1:**
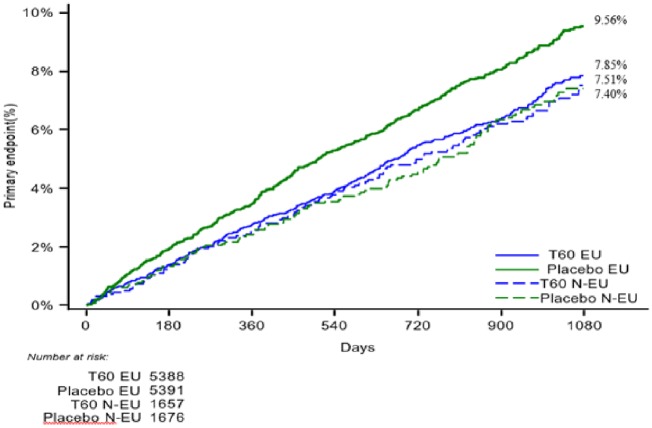
Primary endpoint for ticagrelor 60** **mg vs. placebo, European label and non-European label patients. T60 EU: ticagrelor 60** **mg according to European label. Placebo EU: placebo treatment according to European label. T60 N-EU: ticagrelor 60** **mg to non-European label patients. Placebo N-EU: placebo treatment to non-European label patients.

**Figure 2:**
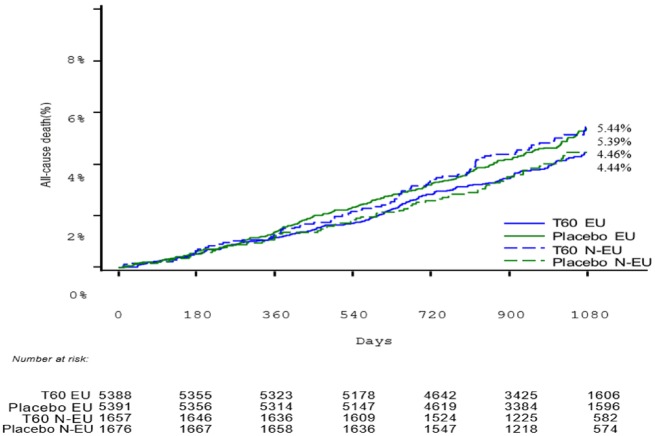
All-cause death for ticagrelor 60** **mg vs. placebo, European label and non-European label patients. T60 EU: ticagrelor 60** **mg according to European label. Placebo EU: placebo treatment according to European label. T60 N-EU: ticagrelor 60** **mg to non-European label patients. Placebo N-EU: placebo treatment to non-European label patients.

In the EU label population, the composite of CV death, MI, or stroke occurred in 373 patients (KM rate 7.9%) in the ticagrelor 60 mg group and in 463 patients in the placebo group (KM rate 9.6%; *Figure 1*); HR 0.80 (95% CI 0.70–0.91; *P* = 0.001), when compared with HR 1.00 (95% CI 0.77–1.30; *P* = 0.98) among the non-EU label population (*P*-value for interaction 0.12). The absolute risk reduction over 3 years was 1.7%, leading to a NNT of 58. In the EU label population, corresponding HRs for the components of the primary composite endpoint were 0.71 (95% CI 0.56–0.90; *P* = 0.0041) for CV death, 0.83 (95% CI 0.70–0.99; *P* = 0.041) for MI, and 0.74 (95% CI 0.55–1.01; *P* = 0.058) for stroke (*Table 2*). The HR for coronary heart disease death was 0.72 (95% CI 0.53–0.97; *P* = 0.03) and for all-cause death 0.80 (95% CI 0.67–0.96; *P* = 0.018) (see *Figure 2*).

**Table 2:** Efficacy of ticagrelor 60 mg vs. placebo in the European label population

Outcomes	Ticagrelor 60 mg bid (*N* = 5388)		Placebo (*N* = 5391)		HR (95% CI)	*P*-value
	Number of events	KM rate (%)	Number of events	KM rate (%)		
Composite of CV death/MI/Stroke	373	7.85	463	9.56	0.80 (0.70–0.91)	0.0011
CV death	119	2.58	167	3.58	0.71 (0.56–0.90)	0.0041
Coronary heart disease death	75	1.59	104	2.15	0.72 (0.53–0.97)	0.0282
MI	230	4.85	274	5.59	0.83 (0.70–0.99)	0.0406
Stroke	71	1.52	95	2.04	0.74 (0.55–1.01)	0.0583
All-cause mortality	206	4.44	256	5.39	0.80 (0.67–0.96)	0.0183

The efficacy results were virtually identical when comparing patients who would qualify for the EU label but were randomized to ticagrelor 90 mg to placebo, with a HR for CV death, MI, or stroke of 0.80 (95% CI 0.70–0.92; *P* = 0.0015) (Supplementary material online, *Table S3*). We also did a further subgroup analysis examining the efficacy of ticagrelor in patients with just one or both of the EMA requirements (MI within 2 years and within 1 year from stopping previous P2Y_12_ receptor inhibitor). Among patients who qualified on both points, the benefit of ticagrelor was most apparent (Supplementary material online, *Table S4*). In patients who qualified on just one of the EMA requirements, the difference between ticagrelor and placebo did not reach statistical significance although the trend towards the benefit is still present.

### Safety

Thrombolysis in myocardial infarction major bleeding occurred in 94 patients (KM rate 2.5%) in the ticagrelor 60 mg group and in 43 patients (KM rate 1.1%) in the placebo group; number needed to harm 76, HR 2.36 (1.65–3.39, *P* < 0.001; *Table 3*), when compared with HR 2.13 (95% CI 1.03–4.43; *P* = 0.04) among non-EU label patients (*P*-value for interaction 0.81). The corresponding HRs for fatal or intracranial bleeding were 1.17 (0.68–2.01; *P* = 0.58) in the EU label subgroup, when compared with HR 1.36 (95% CI 0.41–4.46; *P* = 0.61) among the non-EU label patients. The HR for major bleeding for patients who would qualify for the EU label but were randomized to ticagrelor 90 mg vs. placebo was 2.59 (95% CI 1.81–3.70) (Supplementary material online, *Table S5*).

**Table 3:** Safety of ticagrelor 60 mg vs. placebo in the European label population

Outcomes	Ticagrelor 60** **mg bid (*N* = 5322)		Placebo (*N* = 5331)		HR (95% CI)	*P*-value
	Number of events	KM rate (%)	Number of events	KM rate (%)		
TIMI major bleeding	94	2.46	43	1.14	2.36 (1.65–3.39)	<0.0001
TIMI minor bleeding	49	1.39	15	0.39	3.50 (1.96–6.25)	<0.0001
Fatal bleeding	9	0.29	11	0.33	0.88 (0.37–2.13)	0.7825
Intracranial haemorrhage	23	0.68	18	0.49	1.38 (0.74–2.55)	0.3085
Fatal bleeding or intracranial haemorrhage	27	0.79	25	0.67	1.17 (0.68–2.01)	0.5777

### Net clinical benefit

**Figure 3:**
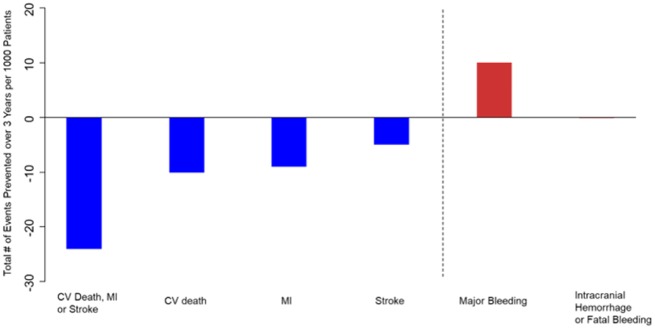
Clinical events prevented and caused per 1000 patients initiated on ticagrelor 60** **mg b.i.d. and followed for 3 years.

The number of events prevented and caused per 1000 patients is shown in *Figure 3*. Treating 1000 patients with ticagrelor 60 mg b.i.d. for 3 years would be expected to prevent 24 major adverse CV events, including 10 CV deaths, 9 MIs, and 5 strokes, while causing 10 major bleeds but no cases of intracranial or fatal bleeding. Thus, the NNT over 3 years to prevent one irreversible event (CV death, MI, stroke, intracranial haemorrhage, or fatal bleeding) was 42.

## Discussion

The present analysis defines the clinical efficacy of dual antiplatelet treatment with 60 mg b.i.d. ticagrelor post-MI when initiated according to the CHMP-EMA approved EU label, i.e. that after the initial year of treatment with 90 mg ticagrelor b.i.d., the patient is shifted to 60 mg b.i.d. without, or with only a briefer interruption.^7^ In such a population, ticagrelor reduced the risk of the primary endpoint of CV death, MI, or stroke by 20%, coronary heart death by 28%, CV mortality by 29%, and all-cause mortality by 20%. Various mechanisms may explain the apparently enhanced benefit of ticagrelor in this population: for example, there is an increased MACE rate in the first 3 months after cessation of P2Y_12_ receptor inhibitor^6^^,^^10^ so ticagrelor-treated patients in the EU label group would have received some protection during this higher-risk period. Furthermore, there was a higher proportion of patients with multivessel coronary artery disease in the EU label group and we have shown these patients to have a greater absolute risk reduction with ticagrelor, including for coronary heart disease-related death, compared to patients with single-vessel disease.^11^ Our analysis aims to guide prescribing clinicians by aiding prediction of the benefit that might be achieved when patients meeting the EU label criteria are switched to ticagrelor 60 mg b.i.d. after 1 year of treatment with ticagrelor 90 mg b.i.d. The US label for ticagrelor is different from the EU label in that it suggests down-shifting from 90 to 60 mg b.i.d. after 12 months of treatment but otherwise has no suggested time limits or time-based guidance.

Any consideration of prolonged antithrombotic therapy must also take safety into account. As expected, prolonged antithrombotic therapy was associated with more bleeding, but fatal or intracranial bleeding was not increased in this population in whom a high risk of life-threatening bleeding had been excluded at enrolment. Thus, in terms of net clinical benefit, for every 1000 eligible patients treated for 3 years, 24 major CV events would be avoided at the cost of only 10 major bleeds. Moreover, there was no excess of fatal or intracranial bleeds, and all-cause mortality was reduced.

Decisions on the safety and effectiveness of a drug in its intended use can be guided by an evaluation of the balance between the benefits and risks. The appropriate approach to such evaluation needs to depend on the severity of the disease and the intervention studied. The full complement of efficacy outcomes and safety evaluations in PEGASUS encompasses a range of event types with varying clinical significance; however, the assessment of the benefit-risk profile of ticagrelor used focuses primarily on those events with the greatest clinical importance. This approach has been supported as the most appropriate one for the assessment of benefit-risk balance since it compares endpoints of similar clinical impact, and integrates clinical judgement supported by quantitative analysis.^9^

While other therapeutic options may be considered such as the combination of low-dose factor Xa treatment^12^ and aspirin that combination has only been reported on patients further away from their index infarction. There are no directly comparative studies between long-term dual antiplatelet treatment with ticagrelor or treatment with low-dose factor Xa inhibitors on top of aspirin.

### Limitations

This is, by definition, a *post hoc* analysis since this subset was defined by regulators and not prospectively. The statistical analysis did not account for multiplicity of testing and this was not a prespecified analysis so therefore *per se* is hypothesis generating. However, the present analysis is of major clinical relevance to physicians and patients since this defines the benefits and risks to be expected in routine clinical practice.

## Conclusions

In PEGASUS-TIMI 54, treatment with ticagrelor 60 mg b.i.d. in patients more recent to their MI or ADP receptor blocker discontinuation, as recommended in the EU label, reduced the risk of CV death, MI, or stroke by 20%, CV death by 29%, and all-cause mortality by 20%. Overall TIMI major bleeding was increased, but fatal or intracranial bleeding were not significantly different from placebo. There appears to be a favourable benefit-risk balance for long-term ticagrelor 60 mg b.i.d. in this population.

## Funding

This study was supported by a grant from AstraZeneca.

**Conflict of interest:** M.D. discloses the following relationships: Advisory board: Novo Nordisk, AstraZeneca, Boehringer Ingelheim, Bayer. Speakers fee: AstraZeneca, Boehringer Ingelheim, Bayer. R.F.S. discloses the following relationships: institutional research grants from AstraZeneca and PlaqueTec; consultancy fees from Actelion, AstraZeneca, Avacta, Bayer, Bristol Myers Squibb/Pfizer, Idorsia, Novartis, PlaqueTec, and Thromboserin; and honoraria from AstraZeneca and Bayer. D.L.B. discloses the following relationships—Advisory Board: Cardax, Elsevier Practice Update Cardiology, Medscape Cardiology, Regado Biosciences; Board of Directors: Boston VA Research Institute, Society of Cardiovascular Patient Care, TobeSoft; Chair: American Heart Association Quality Oversight Committee; Data Monitoring Committees: Baim Institute for Clinical Research (formerly Harvard Clinical Research Institute, for the PORTICO trial, funded by St. Jude Medical, now Abbott), Cleveland Clinic, Duke Clinical Research Institute, Mayo Clinic, Mount Sinai School of Medicine (for the ENVISAGE trial, funded by Daiichi Sankyo), Population Health Research Institute; Honoraria: American College of Cardiology (Senior Associate Editor, Clinical Trials and News, ACC.org; Vice-Chair, ACC Accreditation Committee), Baim Institute for Clinical Research (formerly Harvard Clinical Research Institute; RE-DUAL PCI clinical trial steering committee funded by Boehringer Ingelheim), Belvoir Publications (Editor in Chief, Harvard Heart Letter), Duke Clinical Research Institute (clinical trial steering committees), HMP Global (Editor in Chief, Journal of Invasive Cardiology), Journal of the American College of Cardiology (Guest Editor; Associate Editor), Population Health Research Institute (for the COMPASS operations committee, publications committee, steering committee, and USA national co-leader, funded by Bayer), Slack Publications (Chief Medical Editor, Cardiology Today’s Intervention), Society of Cardiovascular Patient Care (Secretary/Treasurer), WebMD (CME steering committees); Other: Clinical Cardiology (Deputy Editor), NCDR-ACTION Registry Steering Committee (Chair), VA CART Research and Publications Committee (Chair); Research Funding: Abbott, Amarin, Amgen, AstraZeneca, Bayer, Boehringer Ingelheim, Bristol-Myers Squibb, Chiesi, Eisai, Ethicon, Forest Laboratories, Idorsia, Ironwood, Ischemix, Lilly, Medtronic, PhaseBio, Pfizer, Regeneron, Roche, Sanofi Aventis, Synaptic, The Medicines Company; Royalties: Elsevier (Editor, Cardiovascular Intervention: A Companion to Braunwald’s Heart Disease); Site Co-Investigator: Biotronik, Boston Scientific, St. Jude Medical (now Abbott), Svelte; Trustee: American College of Cardiology; Unfunded Research: FlowCo, Merck, PLx Pharma, Takeda. C.H. discloses the following relationships: Advisory Board and speakers fee from AstraZeneca. R.G.K. discloses the following relationships: speakers bureau: Boehringer Ingelheim, Pfizer, Merck, Bayer AG. M.C. discloses the following relationships: speakers bureau and advisory boards: AstraZeneca. J.S. reports no relationships to disclose. A.H. is an employee of AstraZeneca. F.K. reports no relationships. G.K. reports no relationships. A.B. discloses the following relationships: consulting fees from: AstraZeneca, Sanofi-Aventis, Bristol Myers Squibb/Pfizer, GlaxoSmithKline, Bayer, Novartis; investigator fees from: AstraZeneca, Sanofi-Aventis, GlaxoSmithKline, Novartis, Bristol Myers Squibb/Pfizer, Eisai; honoraria for lectures from: AstraZeneca, Sanofi-Aventis, Bristol Myers Squibb/Pfizer, Novartis. P.J. is an employee of AstraZeneca. O.B. is an employee of AstraZeneca. F.V.d.W. and G.M. report no relationships.

## Supplementary Material

pvz020_Supplementary_Data
