# Supplementary material for: Efficacy and safety with ticagrelor in patients with prior myocardial infarction in the approved European label: insights from PEGASUS-TIMI 54
Source: Eur Heart J Cardiovasc Pharmacother. 2019 Jun 20;5(4):200–6. doi: 10.1093/ehjcvp/pvz020 (PMC6749839; doi:10.1093/ehjcvp/pvz020)
Supplement: pvz020_Supplementary_Data [file pvz020_supplementary_data.docx]

**Supplementary Table 1. EU-label vs. non-EU-label population for ticagrelor 60 mg and placebo pooled**

| **Characteristic** | **EU-Label Population** | **Non-EU Label Population** | **P value** |
| --- | --- | --- | --- |
| N | 10779 | 3333 |  |
| Age mean (SD) | 65.2(8.4) | 65.5(8.1) | 0.0341 |
| Female | 2581(23.94%) | 797(23.91%) | 0.9695 |
| White | 9198(85.33%) | 3003(90.1%) | <0.0001 |
| Weight kg, mean (SD) | 81.8(17.0) | 82.5(16.3) | 0.0148 |
| History of Hypertension | 8358(77.55%) | 2587(77.62%) | 0.9317 |
| History of Hypercholesterolaemia | 8301(77.02%) | 2530(75.95%) | 0.2034 |
| Current Smoker | 1804(16.75%) | 545(16.35%) | 0.5937 |
| History of Diabetes | 3484(32.32%) | 1081(32.44%) | 0.8963 |
| Multivessel Coronary Artery Disease | 6613(61.36%) | 1790(53.74%) | <0.0001 |
| History of PCI | 9147(84.88%) | 2569(77.08%) | <0.0001 |
| History of Second Prior MI | 1784(16.55%) | 572(17.17%) | 0.4045 |
| History of PAD | 618(5.73%) | 154(4.62%) | 0.0135 |
| eGRR <60 ml/min/1.73m2 | 2417(22.7%) | 779(23.65%) | 0.2592 |
| Days from P2Y12 discontinuation  median (IQR) | 34 (1, 190) | 588 (466, 719) |  |
| Qualifying Event |  |  |  |
| Years since MI, median (IQR) | 1.5(1.2,1.9) | 2.5(2.3,2.8) | N/A |
| Type of MI |  |  | <0.001 |
| NSTEMI | 4386(40.74%) | 1299(39.07%) |  |
| STEMI | 5800(53.87%) | 1766(53.11%) |  |
| Unknown | 581(5.4%) | 260(7.82%) |  |
| Medications at baseline |  |  |  |
| Aspirin | 10763(99.85%) | 3330(99.91%) | 0.4214 |
| Statin | 10048(93.22%) | 3030(90.91%) | <0.0001 |
| Beta blocker | 8980(83.31%) | 2694(80.83%) | 0.0009 |
| ACE-I or ARB | 8651(80.26%) | 2677(80.32%) | 0.9392 |

**Supplementary Table 2. Baseline characteristics for Ticagrelor 90 mg, EU Label and placebo**

| **Characteristic** | **90 mg bid** | **Placebo** |
| --- | --- | --- |
| N | 5374 | 5391 |
| Age mean (SD) | 65.2(8.4) | 65.3(8.3) |
| Female | 1226(22.81%) | 1314(24.38%) |
| White | 4610(85.33%) | 4606(85.44%) |
| Weight, kg, mean (SD) | 82.1(16.8) | 81.6(16.8) |
| History of Hypertension | 4176(77.71%) | 4175(77.44%) |
| History of Hypercholesterolaemia | 4166(77.52%) | 4179(77.52%) |
| Current Smoker | 891(16.59%) | 865(16.06%) |
| History of Diabetes | 1696(31.56%) | 1710(31.72%) |
| Multivessel Coronary Artery Disease | 3288(61.18%) | 3300(61.21%) |
| History of PCI | 4575(85.13%) | 4563(84.66%) |
| History of Second Prior MI | 866(16.11%) | 900(16.69%) |
| History of PAD | 284(5.28%) | 317(5.88%) |
| eGRR <60 ml/min/1.73m2 | 1230(22.23%) | 1239(23.25%) |
| Qualifying Event |  |  |
| Years since MI, median (IQR) | 1.5(1.2,1.9) | 2.5(2.3,2.8) |
| Type of MI |  |  |
| NSTEMI | 2230(41.53%) | 2177(40.43%) |
| STEMI | 2888(53.78%) | 2928(54.38%) |
| Unknown | 252(4.69%) | 279(5.18%) |
| Medications at baseline |  |  |
| Aspirin | 5366(99.85%) | 5382(99.83%) |
| Statin | 5006(93.15%) | 5049(93.66%) |
| Beta blocker | 4451(82.82%) | 4518(83.81%) |
| ACE-I or ARB | 4363(81.19%) | 4341(80.52%) |

There were no statistically significant differences in baseline characteristics by treatment arm

**Supplementary table 3. Efficacy of Ticagrelor 90 mg vs. Placebo in the EU Label Population**

|  | Ticagrelor 90 mg bid  N=5374 | | Placebo  N=5391 | |  |  |
| --- | --- | --- | --- | --- | --- | --- |
| Outcome | # of Events | KM Rate (%) | # of Events | KM Rate (%) | HR (95% CI) | P value |
| Composite of CV Death/MI/Stroke | 374 | 7.84 | 463 | 9.56 | 0.80 (0.70 ,0.92) | 0.0015 |
| CV Death | 130 | 2.72 | 167 | 3.58 | 0.77 (0.62 ,0.97) | 0.0291 |
| Coronary heart disease death | 69 | 1.41 | 104 | 2.15 | 0.66 (0.49 ,0.90) | 0.0076 |
| MI | 214 | 4.51 | 274 | 5.59 | 0.78 (0.65 ,0.93) | 0.0058 |
| Stroke | 75 | 1.62 | 95 | 2.04 | 0.79 (0.58 ,1.07) | 0.1222 |
| All-cause mortality | 238 | 4.89 | 256 | 5.39 | 0.93 (0.78 ,1.11) | 0.3949 |

**Supplementary table 4. Efficacy for Primary Endpoint for Ticagrelor 60 mg among subsets of the EU Label Population**

| Subgroup * | Ticagrelor 60 mg bid | | | Placebo | | |  |
| --- | --- | --- | --- | --- | --- | --- | --- |
|  | # of events | # of subjects | 3-yr  KM Rate | # of events | # of subjects | 3-yr  KM Rate | HR  (95% CI) |
| Either qualifying MI within 2 years *or* P2Y 12 ADP receptor blocker discontinued within 1 year | 148 | 1833 | 8.8% | 161 | 1821 | 9.6% | 0.90  (0.72-1.13) |
| Qualifying MI within 2 years *and* P2Y 12 ADP receptor blocker discontinued within 1 year | 224 | 3545 | 7.3% | 300 | 3559 | 9.5% | 0.74  (0.62-0.88) |

*21 patients without complete data on one of the 2 variables

P interaction 0.16

**Supplementary table 5. Safety of Ticagrelor 90 mg vs. Placebo in the EU Label Population**

|  | Ticagrelor 90 mg bid  N=5326 | | Placebo  N=5331 | |  |  |
| --- | --- | --- | --- | --- | --- | --- |
| Outcome | # of Events | KM Rate (%) | # of Events | KM Rate (%) | HR (95% CI) | P value |
| TIMI Major bleeding | 98 | 2.59 | 43 | 1.14 | 2.59 (1.81 ,3.70) | <.0001 |
| TIMI Minor bleeding | 48 | 1.26 | 15 | 0.39 | 3.60 (2.01 ,6.43) | <.0001 |
| Fatal bleeding | 5 | 0.12 | 11 | 0.33 | 0.52 (0.18 ,1.50) | 0.2266 |
| Intracranial hemorrhage | 23 | 0.58 | 18 | 0.49 | 1.45 (0.78 ,2.68) | 0.2411 |
